# Heterogeneous data integration methods for patient similarity networks

**DOI:** 10.1093/bib/bbac207

**Published:** 2022-06-13

**Authors:** Jessica Gliozzo, Marco Mesiti, Marco Notaro, Alessandro Petrini, Alex Patak, Antonio Puertas-Gallardo, Alberto Paccanaro, Giorgio Valentini, Elena Casiraghi

**Affiliations:** AnacletoLab - Computer Science Department, Universitá degli Studi di Milano, Via Celoria 18, 20135, Milan, Italy; European Commission, Joint Research Centre (JRC), Ispra (VA), Italy; CINI, Infolife National Laboratory, Roma, Italy; AnacletoLab - Computer Science Department, Universitá degli Studi di Milano, Via Celoria 18, 20135, Milan, Italy; CINI, Infolife National Laboratory, Roma, Italy; AnacletoLab - Computer Science Department, Universitá degli Studi di Milano, Via Celoria 18, 20135, Milan, Italy; CINI, Infolife National Laboratory, Roma, Italy; AnacletoLab - Computer Science Department, Universitá degli Studi di Milano, Via Celoria 18, 20135, Milan, Italy; CINI, Infolife National Laboratory, Roma, Italy; European Commission, Joint Research Centre (JRC), Ispra (VA), Italy; European Commission, Joint Research Centre (JRC), Ispra (VA), Italy; Department of Computer Science, Royal Holloway, University of London, Egham, TW20 0EX UK; School of Applied Mathematics (EMAp), Fundação Getúlio Vargas, Rio de Janeiro Brazil; AnacletoLab - Computer Science Department, Universitá degli Studi di Milano, Via Celoria 18, 20135, Milan, Italy; CINI, Infolife National Laboratory, Roma, Italy; DSRC UNIMI, Data Science Research Center, Milano, 20135, Italy; ELLIS, European Laboratory for Learning and Intelligent Systems, Berlin, Germany; AnacletoLab - Computer Science Department, Universitá degli Studi di Milano, Via Celoria 18, 20135, Milan, Italy; CINI, Infolife National Laboratory, Roma, Italy

**Keywords:** patient similarity networks, biomedical applications, multimodal data, data fusion

## Abstract

Patient similarity networks (PSNs), where patients are represented as nodes and their similarities as weighted edges, are being increasingly used in clinical research. These networks provide an insightful summary of the relationships among patients and can be exploited by inductive or transductive learning algorithms for the prediction of patient outcome, phenotype and disease risk. PSNs can also be easily visualized, thus offering a natural way to inspect complex heterogeneous patient data and providing some level of explainability of the predictions obtained by machine learning algorithms. The advent of high-throughput technologies, enabling us to acquire high-dimensional views of the same patients (e.g. omics data, laboratory data, imaging data), calls for the development of data fusion techniques for PSNs in order to leverage this rich heterogeneous information. In this article, we review existing methods for integrating multiple biomedical data views to construct PSNs, together with the different patient similarity measures that have been proposed. We also review methods that have appeared in the machine learning literature but have not yet been applied to PSNs, thus providing a resource to navigate the vast machine learning literature existing on this topic. In particular, we focus on methods that could be used to integrate very heterogeneous datasets, including multi-omics data as well as data derived from clinical information and medical imaging.

## Introduction

In the last decades, medical research has begun to move from a population-based perspective to a personalized one, often referred to as precision medicine, where patients’ biomedical characteristics are leveraged for diagnosis, prognosis and choice of appropriate treatment [1, 2]. In this context, it is widely accepted that if two patients share similar clinical variables and omics profiles, their clinical outcomes should also be similar. Pairwise similarities between patients have a natural representation as graphs—Patient Similarity Networks (PSN)—where nodes represent patients and edges represent the similarity between patients calculated using their clinical and/or biomolecular features. In this framework unsupervised clustering methods and supervised classification models that leverage similarities between patients have been successfully applied to stratify patients and to predict their phenotype or clinical outcome [3–8]. Representing data as graphs provides several advantages, including interpretability and privacy [9], as patient-specific information cannot be recovered from the similarity measures.

The increasing availability of high-throughput technologies able to generate high-dimensional, distributed biomedical datasets, ranging from multi-omics [8] to imaging [10], clinical and demographic data [11], calls for approaches to mine and aggregate salient information [12] with the ultimate aim of building PSNs integrating such diverse datasets. However, the majority of PSNs that have been proposed are built using only one source of information. At the same time, several methods that can integrate heterogenous sources of information into graph structures have appeared in the past decades in the biomedical and machine learning literature.

In this article, we review existing methods for integrating multiple biomedical data views to construct PSNs. Since the type of data being integrated and the specific integration method must be coupled with an appropriate choice of similarity measure, we will also discuss different similarity measures. Importantly, this paper also reviews methods for integrating information into graph structures that appeared in the machine learning literature but have not yet been used for PSNs. We believe that this will be beneficial for the reader, providing a resource to navigate the vast machine learning literature existing on this topic, and possibly inspire the use and development of novel techniques of data integration for PSNs. Moreover, unlike earlier reviews (see e.g. [8, 13–16]), we focus on methods that may be used for patients’ classification and clustering that integrate not only multi-omics data, but also clinical and image sources.

We propose a taxonomy that groups existing methods for building PSNs into three main categories. ’PSN-fusion methods’ [3, 6, 17] build different PSNs, one for each data source, that are then fused together into a single PSN. ’Input data-fusion’ methods [18–21] combine the different data sources into a single dataset that is then used for building a single PSN. Finally, ’Output-fusion methods’ [22–24] build different PSNs, one for each data source, that are analyzed separately, and results are then combined.

**Figure 1:**
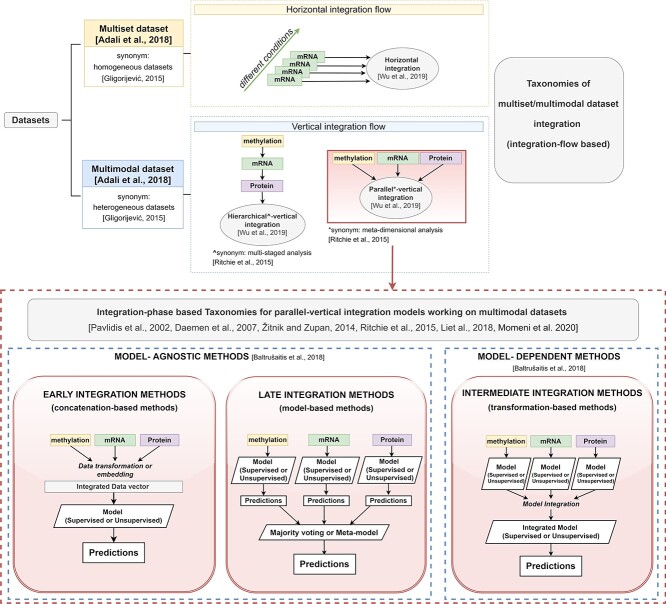
Schema of the main taxonomies proposed in literature for categorizing multimodal integration methods. Considering the data integration flow, literature works identify two broad classes: ’horizontal integration approaches’ and ’vertical integration approaches’. ’Horizontal integration’ approaches fuse ’multisets’ (i.e. datasets where each view is acquired by the same source under different conditions) by independently applying the same process on each view and then pooling the individual results. On the other hand, ’vertical integration approaches’ fuse ’multimodal datasets’ (i.e. datasets composed by semantically different views) through more complex techniques, further categorized as ’hierarchical–vertical integration’ methods and ’parallel–vertical integration’ techniques. The former fuse data views following a ’hierarchy’ driven by biological a priori knowledge whereas the latter do not exploit knowledge-based dependencies between views. ’Parallel-vertical integration’ methods are the most diffused integration methods; they are further classified based on the phase when the data ’integration-step’ is performed with respect to the model construction (red-dashed box). Thus, methods are divided in (1) ’early approaches’, which integrate the data types before model construction, (2) ’late approaches’, which integrate the results of models independently built on each data view and (3) ’intermediate approaches’ where intermediate models are obtained from each view and subsequently integrated. Of note, the latter class of approaches is more dependent on the exploited learning model, which is the reason why they have been also classified as ’model-dependent’ methods opposed to ’model-agnostic’ methods (blue-dashed boxes). We refer interested readers to Appendix A.

Other multimodal data-fusion surveys not specific for PSNs have been proposed in the bioinformatics field by adopting different taxonomies (schematized in Figure 1, Appendix A). Some taxonomies focus on the type of multi-datasets being integrated, thus identifying ’horizontal integration techniques’ [25] (top of Figure 1-yellow box) and ’vertical integration techniques’ [25] (top of Figure 1-light blue box). While the former fuse ’homogeneous multisets’ [26], i.e. ’multimodal datasets’ where each view produces the same data type under different settings, the latter integrate the classic ’heterogeneous’ [26] multimodal datasets. Vertical integration techniques are further classified into methods applying a ’hierarchical (alias ’multi-staged’ [27]) integration’ flow, where ground knowledge about the relationships between the different views is considered during the integration, and methods applying a ’parallel (alias ’meta-dimensional’ [27]) integration’ flow (bottom of Figure 1-red-dashed box), where each view is processed in a similar but independent way. Parallel integration methods are the most diffused in literature given their generalizability. For this reason several reviews concentrate solely on them and introduce taxonomies that distinguish, e.g. ’model-agnostic’ versus ’model-dependent’ methods [28], or exploit an ’early–intermediate–late’ taxonomy [27, 29–33] (described in detail in Appendix A).

Each review paper focuses on different aspects of the multimodal data integration. For example, some works solely focus on integrative unsupervised clustering techniques [34] or supervised multi-omics prediction models [29, 33, 35], or survey data-fusion techniques that are either applied to multi-omics data [16, 25–27, 36], or that apply specific data-fusion techniques (e.g. integrative Bayesian models [13, 37] or multimodal neural networks [38]).

Unlike previous reviews, this work specifically focuses on integrative methods for PSN-based models integrating not only multi-omics data, but also clinical and imaging sources. Each method is critically described to highlight its main advantages and drawbacks, enabling the reader to select the most appropriate approach to answer her/his scientific questions.

Given a set of patients and their corresponding clinical and biomolecular features, the topology of the corresponding PSN depends crucially on how the similarity measure is calculated. Therefore, we begin describing the similarity measurement methods presented in the literature. Our taxonomy of existing methods for building PSNs is described in Sections PSN-fusion methods and Input data-fusion and output-fusion methods. Tables 3–8 summarize the most relevant methods we surveyed.

## PSN construction

The construction of the PSN is a crucial step in PSN analysis models, whose effectiveness mainly depends on the available multimodal datasets from which samples are extracted and on the choice of the measure exploited for pairwise similarity computation between samples.

Several kinds of similarity measures have been adopted in literature for PSN construction: classic distance metrics tailored to the data type [39, 40]; kernel functions [41, 42] that substitute distance metrics; ‘kernels on graphs’ methods [43]. In the remainder, we discuss their characteristics.

The usage of classic ’(opportunely inverted) distances’ or ’similarity metrics’ [39, 40] is often preferred when the data types are normalized and homogeneous. As an example, PSNs on continuous, normalized, data have been constructed by using the cosine similarity [5, 44], or the Euclidean [45] or Mahalanobis distance [45]; PSNs on discrete data types have been built by exploiting the Chi-squared distance [3, 6]; binary data have been handled by using the Jaccard distance [46] or many other distance measures (see [47] for a list of 76 metrics and measures specifically designed for binary data).

When data-blocks with heterogeneous and/or normalized variable types are available, more articulated schemas [6, 48] have been proposed to integrate different similarity metrics into a unique measure. As an example, in [48] the authors proposed a supervised Cox regression model to initially learn a weight for each variable; the learnt weights are then used to compute a similarity score as a weighted sum of individual similarities obtained on each feature by using standard metrics. In this way, different similarity metrics can be used on the different variables based on their type, and the influence of each variable to the global similarity score is weighted on the prediction (e.g. survival time when using a Cox regression model). On the other hand, when dealing with datasets composed by continuous non-normalized variable types, Pai *et al*. [6] propose computing the average of all the normalized similarities over each variable, where the normalization is essentially a min–max normalization.

When dealing with complex problems, literature works often rely on ’Kernel functions’ [49] for PSN computation. The rationale behind this choice is based on the assumption that point separability is often improved after a nonlinear projection of points into a higher-dimensional space. Kernel functions are particularly appealing in this context since they express pairwise distances in a higher-dimensional space by directly using the (lower-dimensional) input samples, therefore avoiding the expensive explicit computation of a nonlinear higher-dimensional mapping followed by pairwise similarity evaluation (using the well-known ’kernel-trick’). Even in this case the choice of the kernel function must be tailored to the data type that is crucial to obtain reliable results. In this context, PSNs are often computed in literature methods working on biomedical data by using classic parametric ’normalized linear kernels’ [30, 50], ’polynomial kernels’ or ’Gaussian kernels’ [51, 52], whose parameters are tuned to optimize performance. As an example, the prognostic approach presented in [30] obtains a set of unimodal PSNs by applying normalized linear kernels on each of the data sources containing clinical and multi-omics datasets. In this case, the usage of the same kernel function on different sources is appropriate because they are characterized by the same data type (real-valued data type).

In a subsequent work [53], the same authors extend the dataset by including categorical and integer data types; therefore, they substitute the linear kernels with a set of kernels tailored on each data type being processed. Of note, the kernels used in [30, 53] are always normalized. This is a crucial characteristic when integrating multiple kernels because comparable kernel scales are obtained, therefore facilitating the kernel integration. Moreover, in the case of kernel-aggregation systems exploiting weighted averages of the unimodal kernels, normalization also improves the interpretability of the computed integration weights, the latest being directly related to the importance of their respective kernel [53].

A recent advance in the field of PSN analysis is provided by unsupervised methods that compute the PSN through the ’scaled exponential Euclidean kernel’ [3] and its modifications [54, 55]. They essentially apply a local normalization of the distance between a central node and any of its neighbors, so that distances are independent from the neighborhood scales. Their application in the context of unsupervised patient clustering through PSN analysis has obtained promising results [3] (see Section SNF-based methods).

Given its effectiveness, the scaled Euclidean distance has been extended in [54] to deal with heterogeneous data types containing continuous and boolean variables. More precisely, the similarity on boolean data is measured by using the weighted Hamming distance with weights computed by supervised approaches or pre-set based on existing knowledge. Further, in [55] the authors propose adopting the Chebyshev distance instead of the Euclidean distance.

Gliozzo et al. [7] extend to PSNs a previous ’kernel-based’ approach originally applied to the semi-supervised analysis of biomolecular networks [56]. More precisely, the authors obtain promising outcome predictions on unimodal PSNs by firstly using the filtered Pearson correlation (by setting to zero all negative values) to measure similarities between unimodal gene expression profiles, and then applying a random walk kernel to strengthen high similarities while diminishing low ones. The neighborhoods identified in the obtained PSN are then used to compute a score for each patient, which is thresholded to obtain the desired classification. While unimodal PSNs are exploited in [7], the works proposed in [57] and [58] exploit random-walks to compute similarities in a multimodal setting.

To improve informativeness, Tables 1 and 2 sketch the similarity measures/ methods used for PSN construction by notable literature works exploiting multimodal datasets; for each paper we report the data types of the different data sources exploited for the investigation, and the similarity measures/methods used for building the corresponding unimodal PSNs.

**Table 1:** Similarity measures/methods used in literature to build PSNs. For notable works in literature the table reports: the reference of the literature work presenting a multimodal PSN analysis method (column ’References’), the data types (column ’Data type’) of the different sources (column ’Data’) used for the investigation, and the similarity measures/methods exploited for building the unimodal PSNs

**References**	**Data type**	**Data**	**Similarity measure/method**
[46]	Binary	ICD-9 diagnosis code	Jaccard similarity
[44]	Continuous,	Clinical data	Cosine similarity
	Categorical, discrete		
[5]	Continuous	Clinical data	Cosine similarity
	Categorical, discrete		
[61]	Continuous	mRNA, PPI	Pearson correlation
[7]	Continuous	mRNA	Pearson correlation
[6]	Continuous	Clinical variables	Mean of normalized difference
		Individual gene	Normalized difference
		Genes in pathways/networks	Pearson correlation
	Discrete	Categorical-ordinal variable	Normalized difference
		(e.g. tumor stage)	
		Unbinned counts	Shared incidence
		(e.g. mutation data)	in a grouped unit
		Matrix scores	chi-square distance
		(e.g. response to questionnaire)	
[3]	Continuous	mRNA, miRNA, DNA methylation	Scaled exponential kernel of Euclidean distance
	Discrete		chi-squared distance
	Binary		agreement-based measure
[54]	Continuous binary	mRNA, DNA methylation somatic mutation	Scaled exponential kernel of weighted Euclidean distance scaled exponential kernel of weighted Hamming distance
[62]	Continuous	mRNA, miRNA, DNA methylation	Scaled exponential kernel of Euclidean distance
[63]	Categorical, discrete	Demographic, APOE4 allele status,	squared-exponential kernel
		MRI	
[55]	Continuous	Gene expression,	Kernel of Chebyshev distance
		miRNA,	
		Isoform expression	
[48]	Continuous, categorical, discrete	Clinical data	Weighted sum of distances
			with weight determined by a scaled
			Cox regression coefficient

**ICD-9**: International Classification of Diseases Version 9; **CNV:** copy number variation; **miRNA:** micro RNA; **MRI:** magnetic resonance imaging; **mRNA:** messenger RNA; **PPI:** protein–protein interaction

**Table 2:** Similarity measures/methods used in literature to build PSNs. For notable works in literature the table reports: the reference of the literature work presenting a multimodal PSN analysis method (column ’References’), the data types (column ’Data type’) of the different sources (column ’Data’) exploiting for the investigation, and the similarity measures/methods exploited for building the unimodal PSNs.

**Reference**	**Data type**	**Data**	**Similarity measure/method**
[30, 53]	continuous	mRNA, clinical	normalized linear kernel
	categorical, discrete, binary	clinical	
[64]	discrete	MRI	gaussian kernel
	continuous	CSF	
[51]	continuous	mRNA, miRNA, CNV,	gaussian kernel
		DNA methylation,	
	discrete	clinical	
[50]	continuous,	mRNA, miRNA, CNV,	linear kernel
		DNA methylation, RPPA,	
	binary, discrete	somatic mutations, clinical data	
[65]	continuous	mRNA, CNV, DNA methylation	normalized linear kernel,
			normalized polynomial kernel,
			normalized gaussian kernel
[53]	continuous, categorical (ordinal)	clinical variables	absolute difference of values/ranks of two subjects compared and rescaled using variable range
	categorical (nominal)	clinical variables	kernel defined using Kronecker delta function
[57]	continuous, binary	mRNA, RPPA, somatic mutation	novel graph kernel called SmSPK

CNV: Copy Number Variation; **miRNA**: micro RNA; **mRNA**: messenger RNA; **RPPA**: Reverse-Phase Protein Arrays; **CSF**: CerebroSpinal Fluid.

Even if a wide range of similarity computation methods has been proposed in literature, a consensus on which strategy performs better on specific data types and problems in the context of precision medicine is still lacking. Some tentative experiments have been conducted for determining the best-performing strategies (see e.g. [59, 60]), but the lack of common benchmark datasets prevents an unbiased comparison of the different proposed approaches.

## PSN-fusion methods

**Figure 2:**
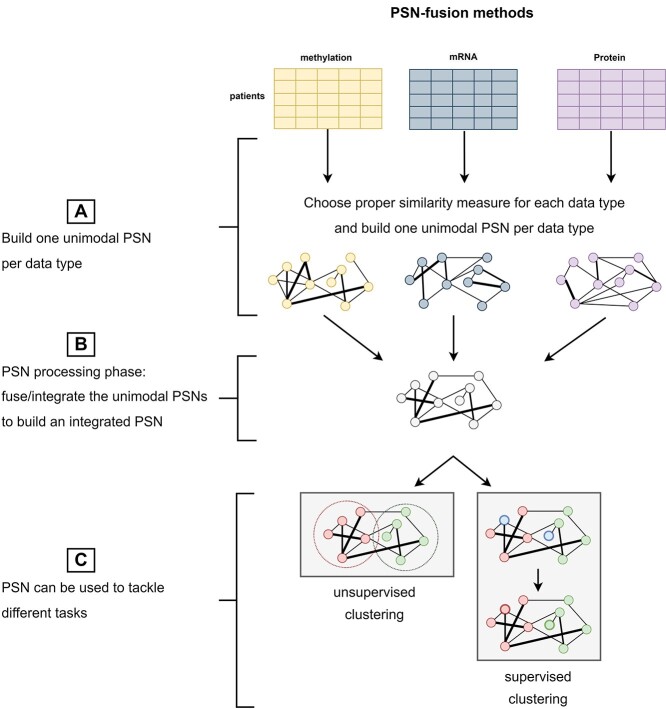
High-level representation of PSN-fusion methods. (**A**) Given a set of matrices, each representing the patients vectors acquired from one source, proper similarity measures or kernel functions are used to build a set of unimodal PSNs (one PSN per data source or data type); (**B**) all the PSNs are then fused through either MKL methods, SNF methods or other PSN-fusion approaches; (**C**) the integrated PSN is processed either by unsupervised clustering algorithms for solving, e.g. patients’ subtype prediction tasks, or by supervised classifier models for, e.g. patients’ outcome prediction.

PSN-fusion methods have been specifically developed to process a set of unimodal PSNs and produce an integrated PSN. In Figure 2 we sketch the generic workflow of the PSN-fusion methods. They start by building unimodal PSNs on each data source or data type (Figure 2**A**). Mind that the choice of the similarity measure/kernel function used to build each PSN (Section PSN construction) is crucial for obtaining informative unimodal PSNs, which would otherwise hamper the achievement of successful results. Next, the aggregation of the unimodal PSNs (Figure 2**B**) is performed by either Multiple Kernel Learning methods (MKL, Section MKL-based methods, Table 3), which run optimization algorithms inherited from the machine learning field to find the optimal weights of an additive unimodal kernel aggregation, or approaches stemming from the seminal Similarity Network Fusion algorithm (SNF—[3], Section SNF-based methods, Table 4), which use different strategies to diffuse the similarity information both between neighboring nodes in each unimodal PSN and between corresponding nodes in different PSNs, or other network-based approaches (Section Other PSN-fusion methods and Table 5).

**Table 3:** MKL-based PSN-fusion methods. For each method, the table reports: the name/acronym with the corresponding reference paper; whether it requires the same set of patients across all data modalities (i.e. ‘Matched Samples’); the dataset used to develop and evaluate the approach in the reference paper and the corresponding sample cardinality and data types composing the dataset; the exploited integration method; the application task and the code availability (with link to the repository and programming languages for which the code is available)

Name	Matched samples	Dataset	Sample cardinality	Data type	Integration approach	Task	Code and Language
PAMOGK$^{1}$	x	TCGA KIRC	361	Somatic mutation	MKKM	Unsupervised Clustering	MATLAB,
[57]		NCI-PID at NDEXBio		mRNA	[77]	(Patient subtype identification)	Python code
				RPPA			
[64]	x	ADNI	120	CSF features	MKL	Supervised Classification	
				MRI	[17]	(HC versus MCI patients)	
[69]	x	TCGA	585	Histopathological images	simpleMKL	Supervised Classification	
				Clinical data	[17]	(Patient’s Prognosis)	
				mRNA			
				methy			
				RPPA			
[67]	x	TCGA GBM	125	Histopathological images	simpleMKL	Supervised Classification	
				CNV	[17]	(Patient’s Prognosis)	
				mRNA			
				miRNA			
MK-FDA [73]	x	Protein dataset	Not provided	Protein sequences	MKL	Supervised Multiclass Classification	
[75]						(Protein subcellular localization)	
[50]	x	14 TCGA datasets	3382	Germline variants	MKL	Supervised Classification	
				Somatic mutation		(Patient’s Survival)	
				CNV			
				mRNA			
				miRNA			
				methy			
[52]	x	TCGA from	989	mRNA	MKL	Unsupervised Clustering	
		mixOmics		miRNA		(Patients’ subtype identification)	
				methy			
rMKL-LPP	x	TCGA GBM	213	mRNA	MKL	Unsupervised Clustering	
[76]		TCGA BIC	105	miRNA		(Patient subtype identification)	
		TCGA KIRC	122	methy			
		TCGA LUSC	106				
		TCGA COAD	92				

**ADNI**: Alzheimer’s Disease Neuroimaging Initiative; **CNV**: Copy Number Variation; **CSF**: CerebroSpinal Fluid; **HC**: Healthy Control; **MCI**: Mild Cognitive Impairment; **methy**: DNA methylation; **miRNA**: micro RNA; **MKKM**: Multiple kernel k-means clustering; **MKL**: Multiple Kernel Learning; **MRI**: Magnetic Resonance Imaging; **mRNA**: messenger RNA; **NCI-PID**: National Cancer Institute—Pathway Interaction Database; **RPPA**: Reverse-Phase Protein Arrays; **TCGA+cancer code**: The Cancer Genome Atlas+ *link to complete cancer codes*.

**Table 4:** SNF-based PSN-fusion methods. For each method, the table reports: the name/acronym with the corresponding reference paper; whether it requires the same set of patients across all data modalities (i.e. ‘Matched Samples’); the dataset used to develop and evaluate the approach in the reference paper and the corresponding sample cardinality and data types composing the dataset; the exploited integration method; the application task and the code availability (with link to the repository and programming languages for which the code is available)

Name	Matched samples	Dataset	Sample cardinality	Data type	Integration approach	Task	Code and Language
SNF [3]	x	TCGA GBM	215	mRNA	SNF	Unsupervised Clustering	MATLAB code, R code
				miRNA		(Patient subtype identification)	
				methy			
ANF [87]	x	TCGA LUSC	2193	mRNA	SNF	Unsupervised Clustering	R code
		TCGA Adrenal		miRNA		(Patient subtype identification)	
		TCGA Gland		methy			
		TCGA KIRC					
		TCGA Uterus					
HSNF [89]	x	TCGA BIC	105	mRNA	SNF	Unsupervised Clustering	
		TCGA GBM	215	miRNA		(Patient subtype identification)	
		TCGA KIRC	122	methy			
		TCGA LUSC	106				
		TCGA COAD	92				
SKF [90]	x	TCGA BIC	1071	mRNA	SNF	Unsupervised Clustering	MATLAB code
		TCGA COAD	426	miRNA		(Patient subtype identification)	
		TCGA KIRC	868	isoform level			
		TCGA LUSC	981				
		TCGA Stomach	377				
ab-SNF [54]	x	TCGA LIHC	Not provided	somatic mutation	SNF	Unsupervised Clustering	R code
		TCGA KIRP		mRNA		(Patient subtype identification)	
		TCGA BIC		methy			
NEMO [93, 94]		TCGA AML	3168 across	mRNA	SNF	Unsupervised Clustering	R code
		TCGA BIC	all datasets	miRNA		(Patient subtype identification)	
		TCGA COAD		methy			
		TCGA GBM					
		TCGA KIRC					
		TCGA LIHC					
		TCGA LUSC					
		TCGA SKCM					
		TCGA OV					
		TCGA SARC					

**methy**: DNA methylation; **miRNA**: micro RNA; **mRNA**: messenger RNA; **SNF**: Similarity Network Fusion; **TCGA+cancer code**: The Cancer Genome Atlas+ *link to complete cancer codes*.

**Table 5:** Other PSN-fusion methods. For each method, the table reports: the name/acronym with the corresponding reference paper; whether it requires the same set of patients across all data modalities (i.e. ‘Matched Samples’); the dataset used to develop and evaluate the approach in the reference paper and the corresponding sample cardinality and data types composing the dataset; the exploited integration method; the application task and the code availability (with link to the repository and programming languages for which the code is available)

Name	Matched samples	Dataset	Sample cardinality	Data type	Integration approach	Task	Code and Language
netDx [6]	x	TCGA KIRC	150	mRNA	Average score	Supervised classification	R code
		TCGA OV	252	miRNA		(patient’s survival)	
		TCGA GBM	155	methy			
		TCGA LUSC	77	CNV			
				RPPA			
				clinical data			
RWRF, RWRNF [58]	x	TCGA ACC	76	mRNA	RWR	Unsupervised clustering	R code
		TCGA BLCA	396	miRNA		(patient subtype identification)	
		TCGA HNSC	469	methy			
		TCGA UVM	80				
		TCGA PAAD	175				
		TCGA THCA	492				
MRF-MSC [95]	x	TCGA COAD	92	mRNA	Maximization	Unsupervised clustering	
		TCGA GBM	215	miRNA	of alignment to all	(patient subtype identification)	
		TCGA BRCA	105	methy	the unimodal PSNs		
		TCGA KIRC	122				
		TCGA LSCC	106				

**CNV**: Copy Number Variation; **methy**: DNA methylation; **miRNA**: micro RNA; **mRNA**: messenger RNA; **PSN**: Patient Similarity Network; **RPPA**: Reverse Phase Protein Array; **RWR**: Random Walk Kernel with Restart; **TCGA+cancer code**: The Cancer Genome Atlas+ *link to complete cancer codes*.

The integrated PSN may be finally used as input to unsupervised clustering methods aiming at, e.g. identifying patients’ subtypes, or supervised classification methods predicting, e.g. patients’ risk, prognosis or outcome (Figure 2**C**).

### MKL-based methods

Inheriting theories and algorithms from the machine learning fields, MKL methods [17, 64–66] view the unimodal PSNs as kernels and propose their optimal additive combination, as a weighted sum of the available unimodal kernels. In this context, ‘optimality’ refers to either a ’supervised’ setting or an ’unsupervised’ one.

’Supervised MKL’ algorithms (e.g. simpleMKL [17]) exploit a supervised classifier model designed to work on the fused kernel. Supervision is guaranteed by the availability of a training set composed of samples whose labels are known. Such training set is used by the chosen supervised MKL method to solve a constrained optimization problem that finds the kernel weights and classifier hyper-parameters maximizing the classification accuracy on the training set. On the other side, ’unsupervised MKL’ methods make no use of labeled samples, but instead solve an optimization problem to find the weights that essentially lead to the maximum alignment between the integrated kernel and any of the input unimodal kernels.

Recent PSN-fusion methods exploiting a ’supervised MKL’ strategy are those presented by [30, 50, 53, 64, 67]. The work proposed in [50] designs specific kernels for each omic type in the The Cancer Genome Atlas (TCGA) cancer dataset and then computes the kernel weights by using the training set to optimize the fit of a Cox-survival model.

All the other works [30, 53, 64, 67] share the use of the kernelized Support Vector Machine (SVM) classifiers [68], opportunely modified as defined in [17] and [66] to work on the kernel resulting from an optimal additive sum. In particular, the works proposed by Daemen *et al*. [30, 53] aggregate specific kernels on each clinical data type and uses a classic SVM optimization strategy to derive the optimal weights, while the works proposed in [64] and [67] use the easyMKL algorithm to optimize an svm aggregating multiple kernels defined over multimodal datasets also including opportunely coded imaging sources. More precisely, in [64] authors use the same Gaussian kernels to process both the real cerebrospinal fluid (CSF) biomarkers features and the shape and texture features extracted to code magnetic resonance images (MRI). On the other side, the work proposed in [67] improves upon the work presented in [69] and defines specific kernels for the multi-omics data from the TCGA cancer dataset and for the features automatically extracted from histopathological images (Table 3). The effectiveness of the simpleMKL strategy is witnessed by its several extensions (easyMKL [70], SEMKL [71], SpicyMKL [72]).

As expected, our literature search highlighted that SVMs are the most widely used base-learner models in conjunction with MKL in the context of biomedical predictions; however, some authors have also presented MKL methods using Multiple Kernel Fisher Discriminant Analysis (MK-FDA [73]) or Kernel Regularized Discriminant Analysis [74] as base learners where the single kernel is substituted by multiple kernels. Though these strategies have not been applied on patients’ data, their promising results on the protein subcellular localization prediction task [73, 75] suggest they could be good options for developing a multimodal PSN analysis task.

’Unsupervised MKL’ approaches are described in the works of [52, 76, 77]. The regularized MKL with Locality Preserving Projection algorithm (rMKL-LPP [76]) is an unsupervised, regularized MKL-based clustering approach for the identification of cancer subtypes from multi-omics data. It builds upon the MKL-DR model proposed in [78] to constrain the optimization problem by handling the ‘small-sample-size’ problems caused by the high dimensionality of the input data sources and exploits the theories at the base of the LPP algorithm [79] to find the integrated kernel in a lower-dimensional space that maintains the local neighborhoods relationships. In other words, the model minimizes a function that allows finding both the hyper-parameters of the multiple kernels and their combination weights so that patients that are similar according to ‘many’ input sources (kernels) remain neighbors in the integrated kernel. Further, to avoid restricting the usage of only one kernel per data source or data type, authors add a constrained regularization that avoids overfitting, so that multiple kernels can be used for each source without risking to overfit the data. Similar topological constraints are used by [52] to compute kernel weights such that the resulting integrated kernel maintains the neighborhood relationship described above, and at same time maximizes the alignment (similarity) to all the input kernels.

By contrast, Liu *et al*. [77] leverage the standard kernel k-means clustering [80], which applies k-means in the kernel space, to a ’multiple kernel k-means clustering’ (MKKM) that considers the relationships between all the input kernels. The optimal clusters are found by minimizing a loss that measures the intraclass sample distance as a function of the cluster assignment matrix and the kernel weights. However, differently from other multiple kernel clustering models, the MKKM loss function includes a term that promotes the choice of higher weights for uncorrelated kernels.

### SNF-based methods

PSNs are similarity graphs by definition; therefore, recent promising works apply graph-based algorithms and theories to integrate them. In particular, some authors simply integrate the information from different similarity graphs by using graph kernels [57] or by averaging [58, 81].

On the other side, SNF [3] exploits a nonlinear message-passing algorithm [82] that diffuses the information between all the unimodal PSNs constructed on each data-block until they converge to the integrated PSN. The diffusion process is designed so that the similarity between any two points computed over a specific source is updated and diffused if the two points are neighbors or share common neighbours in the other modalities. SNF has proven to be successful when compared with relevant PSN-fusion methods [83] in the unsupervised clustering task on three real, complex, multi-omics datasets (murine liver—BXD [84], platelet reactivity [85] and Breast Cancer dataset from TCGA—BRCA [86]).

Several works extended SNF in different ways, thus creating a group of algorithms (called SNF-based methods). As an example, Affinity Network Fusion (ANF) [87] has been developed to diminish the computational costs of SNF, by reducing the iterative integration strategy of SNF to a unique step. To this aim, authors design a multigraph where each layer corresponds to a source-specific PSN, and then apply the one-step random walk kernel, where user-defined parameters are the transition probabilities between different layers, and the PSN for a specific layer represents the transition probabilities between nodes in that layer. When tested on multiple TCGA datasets, ANF outperforms SNF both in terms of clustering efficacy and computational costs.

By taking into account that the Euclidean distance metric employed in SNF suffers the curse of dimensionality [88] and may affect the results, [89] presented HSNF (hierarchical SNF), which essentially runs SNF several times, where each iteration uses a set of unimodal PSNs, generated on each data-block by using a randomly sampled feature set. At each iteration, the computed PSNs are fused with the integrated network computed in the precedent steps through SNF. The method is evaluated by its capacity to identify cancer subtypes by applying spectral clustering on the integrated matrix. Though outperforming SNF on several cancer datasets, HSNF has a higher computational cost because of the iteration of SNF.

To reduce noise in the integrated network, the Similarity Kernel Fusion algorithm (SKF) [90] multiplies the PSN built by using SNF with a matrix of weights, where the weight is higher if two samples are included in each other neighbourhood. Moreover, different from SNF, a term in the iterative update function is added to control the amount of information to be retained from the integrated kernel at the preceding step. When compared with SNF and to a simple average fusion of different kernels, SKF obtains comparable or even better performance in the discovery of cancer subtypes from real cancer datasets.

The association-signal-annotation boosted similarity network fusion (ab-SNF) method [54] tries to improve SNF by considering a weighted version of distance measures with the goal to upweight signal features and downweight noisy ones. In this work, the weight for continuous variables consists in a *P*-value computed by the univariate *t*-test to assess the feature significance in predicting the outcome variable; the weights for binary features, such as mutation data, are obtained by considering prior knowledge from databases (e.g. 1 for features related to cancer and 0 otherwise). Given the computed weights, the unimodal PSNs are obtained by using the scaled exponential kernel [3], where the Euclidean distance is substituted by the weighted Euclidean distance, for continuous variables, or the weighted Hamming distance, for binary variables. The use of feature-level weights leads to superior performance in clustering accuracy with respect to SNF on both simulated and real data, whereas subtypes captured by ab-SNF are significant in terms of patient survival on real cancer data.

### Other PSN-fusion methods

NetDx [6] fuses unimodal PSNs by a simple weighted network sum, where the weights for each network are identified by ridge regression to a target network constructed on the training patients in order to enforce higher similarities between positive nodes and lower similarities between nodes belonging to different classes.

Some recent integration methods propose integrating the different PSNs by using a graph-based construction and then compute integrated similarities by visiting the graph through random walk kernels. As an example, [58] propose computing similarities over a multiplex graph composed by a collection of PSNs (layers) each built on an individual data-block. The different layers share the same set of nodes [91], and corresponding nodes in different layers are connected to guarantee connectivity across multiple layers, but are considered as different entities to avoid disrupting the difference between the multiple views available for each node/sample. Then, authors use the random walk kernel with restart (RWR) [92] to express the similarities as the probabilities of reaching a node in a specific layer when another node in the same or in another layer is used as the starting point of the walk. To account for multimodality, that is with the presence of multiple layers, the probability of ‘jumping’ to another layer during the walk is weighted by a parameter $\lambda $. The probabilities are computed by an iterative process that continues until a stationary point is reached. RWRNF [58] is an extension of this method that allows connecting multiple layers by also using edges between neighbourhoods of corresponding nodes. The use of many random walks, starting from all the nodes in each layer, adjusts the weights of the multiplex network taking into account its global topology. Finally, an integrated similarity network is computed by averaging corresponding weights across different layers of the network.

The efficacy provided by the use of similarities computed across local neighborhoods is proven by its use in simpler unsupervised PSN analysis methods. As an example, NEMO (NEighborhood based Multi-Omics clustering, [93, 94]) is an unsupervised clustering approach where authors use a scaled normalized euclidean kernel to compute similarities, which are then made symmetric in a way very similar to SNF and are designed to have values equal to zero for nodes that are not neighbors. Extensive experiments on simulated and real datasets showed the competitive effectiveness and efficiency of NEMO with respect to nine state-of-the-art methods among which one MKL-based method, a spectral clustering method, the classic k-means clustering approach and six clustering methods exploiting an input data-fusion approach (Section Input data-fusion and output-fusion methods).

Finally, a noteworthy PSN-fusion method applied for unsupervised patient subtype identification in the TCGA dataset is Multi-view Spectral Clustering Based on Multi-smooth Representation Fusion (MRF-MSC) [95]. MRF-MSC starts by individually processing each data-block to obtain a smoothed similarity matrix where strong/weak similarities are strengthened/eliminated; this is obtained by solving a regularized optimization problem that computes the similarity matrix in a feature space that minimizes the point-reconstruction error while strengthening the point groupings. Next, a fused similarity matrix that minimizes the weighted distance from all the smoothed source-similarity matrices is obtained by integrating a self-weighting method [96] into the distance minimization problem. Finally, the clusters in fused similarity networks are strengthened by applying the constrained Laplacian rank method and Spectral clustering is then applied to solve the clustering problem.

## Input data-fusion and output-fusion methods

**Figure 3:**
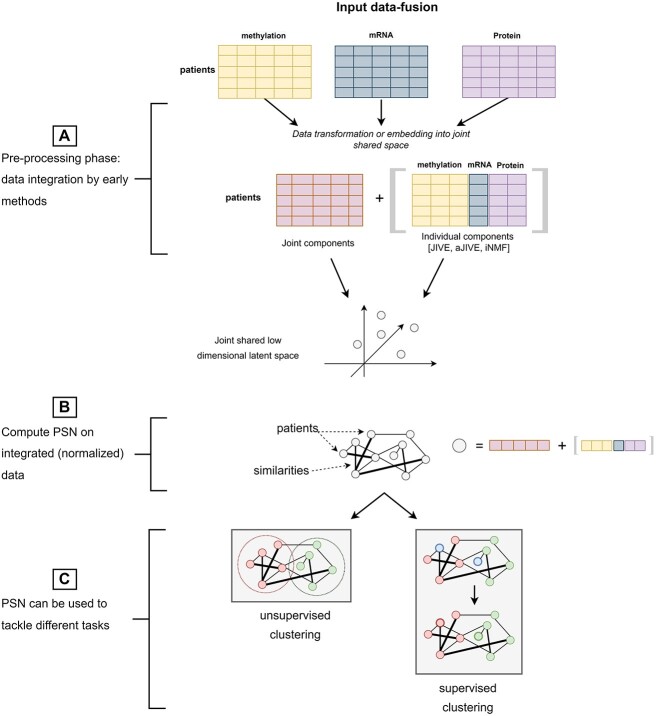
Input data-fusion. (**A**) During the preprocessing phase the data are integrated by either a PCA-based integrative model or a MF-based model. They estimate a shared latent space where the integrated, normalized point representations express the joint structure underlying all the data blocks plus, eventually, the individual structures characterizing each data block (e.g. JIVE [18], aJIVE [97], iNMF [98]); (**B**) a PSN model is then constructed on the integrated profiles by using a classic similarity measure; (**C**) a clustering or supervised classification model is applied to the computed PSN.

**Figure 4:**
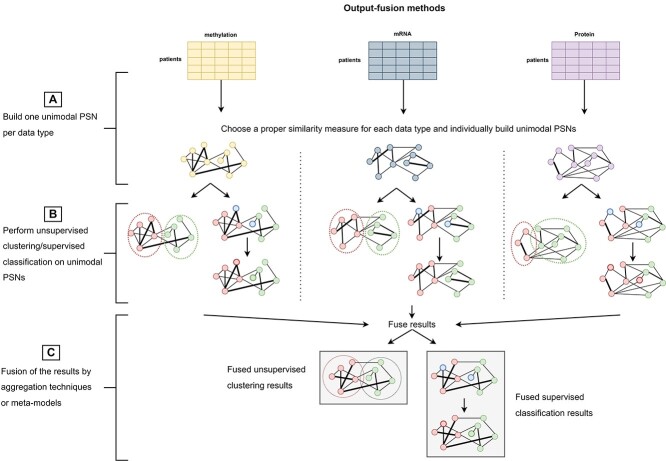
Output-fusion. (**A**) Unimodal PSNs are constructed for each data type or data source and (**B**) each one is individually processed to identify clusters or to classify unknown samples; subsequently, (**C**) a simple aggregation technique or a meta-model is used to obtain the fused/consensus clustering/classification result.

Opposite to PSN-fusion models, the input data-fusion and the output-fusion techniques reviewed in this section integrate the information available either in the multimodal input data (’input data fusion’ methods—Figure 3) or in the output computed by a set of individual unimodal PSN-analysis models (’output fusion’ methods—Figure 4).

’Input data fusion’ methods are schematized in Figure 3. These approaches are based on the assumption that the input samples originally lied in a latent (eventually orthogonal) space from which the multiple source-views have been generated by unknown projections. This results in data-blocks being expressed into separate source-specific spaces that are characterized by: (1) an individual source-specific structure generating an individual variability within each data-block; (2) a joint sample-specific structure [18] resulting in shared variance (collinearities) between data-blocks. Therefore, input data-fusion methods estimate the embedding that back-projects the input data-blocks into a shared latent space minimizing redundancy between the data-blocks while maximizing the individual data-block variability. In other words, all the methods find the ’joint components’ (Figure 3) allowing to capture the greatest amount of shared variance; most of the methods also define ways to identify the ’individual components’ capturing the source-specific variability (Figure 3).

Depending on the technique used to project the data into the shared latent space, we can distinguish input data-fusion methods into PCA-based techniques (Table 6) or Matrix Factorization (MF) or Blind Source Separation (BSS)-based methods (Table 7). One advantage of solving the information-fusion in a preprocessing phase, i.e. preceding the construction of an integrated PSN, is that a standard unimodal PSN-analysis model can be subsequently applied (Figure 3**B**) to deal with clustering or supervised classification problems (Figure 3**C**). In particular, the input data-fusion methods make the choice of the similarity measure to be used for PSN construction particularly easy, since they compute normalized, a-dimensional, integrated point representations, whose pairwise similarities could be handled by classic measures such as the cosine similarity or the inverted euclidean distance. Moreover, a side-effect of the estimated embedding is that the estimated component loadings or factors may be analyzed for uncovering hidden relationships between variables (data analysis task in Tables 6 and 7 and in Figure 3).

**Table 6:** PCA-based and CCA-based input data-fusion methods. For each method, the table reports: the name/acronym with the corresponding reference paper; whether it requires the same set of patients across all data modalities (i.e. ‘Matched Samples’); the dataset used to develop and evaluate the approach in the reference paper and the corresponding sample cardinality and data types composing the dataset; the exploited integration method; the application task and the code availability (with link to the repository and programming languages for which the code is available)

Name	Matched samples	Dataset	Sample cardinality	Data type	Integration approach	Task	Code and Language
CPCA [99]	x	Simulated	Not provided	Numeric	PCA	Data Analysis	
CPCA for missing data [100]	x	Human Mortality Database	143	exposure-to-risk	PCA	Data Analysis	
		(Italy + Switzerland)					
JIVE [18]	x	TCGA BIC	348	mRNA	PCA	Unsupervised Clustering (Patient subtype identification)	R code
				miRNA			
				methy			
				RPPA			
aJIVE [97]	x	TCGA extract from	616	mRNA	PCA	Data Analysis and	R code
		[101]		miRNA		Unsupervised Clustering	
				somatic mutation		(Patient subtype identification)	
				CNV			
				RPPA			
MCCA [102]	x	DLBCL Dataset	203	mRNA	CCA	Data analysis	R code
		[103]		array CGH measurements			
RGCCA [104]	x	SCA Dataset	67 SCA +	pons volume	CCA	Data Analysis	RGCCA/SGCCA
SGCCA [19]		Private	35 Healthy	metabolic features			R code
[105]							
DIABLO [20]	x	TCGA COAD	92	mRNA	(SG)CCA	Data Analysis and	R code
		TCGA KIRC	122	miRNA		Supervised Clustering	
		TCGA GBM	213	methy		(Patient’s Survival)	
		TCGA LUSC	106				
		TCGA BRCA	989				

**CCA**: Canonical Correlation Analysis; **CGH**: Comparative Genomic Hybridization; **CNV**: Copy Number Variation; **DLBCL**: Diffuse Large B-Cell Lymphoma; **methy**: DNA methylation; **miRNA**: micro RNA; **mRNA**: messenger RNA; **PCA**: Principal Component Analysis; **RPPA**: Reverse Phase Protein Array; **SCA**: SpinoCerebellar Ataxia; **(SG)CCA**: (Sparse Generalized) Canonical Correlation Analysis; **TCGA+cancer code**: The Cancer Genome Atlas+ *link to complete cancer codes*.

**Table 7:** MF-based input data-fusion methods. For each method, the table reports: the name/acronym with the corresponding reference paper; whether it requires the same set of patients across all data modalities (i.e. ‘Matched Samples’); the dataset used to develop and evaluate the approach in the reference paper and the corresponding sample cardinality and data types composing the dataset; the exploited integration method; the application task and the code availability (with link to the repository and programming languages for which the code is available). Of note, DFMF [31] has not been applied to patients' data but it could be easily adapted to this end.

Name	Matched samples	Dataset	Sample cardinality	Data type	Integration approach	Task	Code and language
MFA		Brain Cancer	Not provided	Multi-omics	MF	Data Analysis	R code
[107]		Dataset Private					
jNMF	x	TCGA OV	385	mRNA	NMF	Data Analysis	R code
[121]				miRNA			
				methy			
iNMF	x	TCGA OV	592	mRNA	NMF	Unsupervised Clustering	Python code
[98]				miRNA		(Patient subtype identification)	
				methy			
iGMFNA	x	TCGA CHOL	45	mRNA	NMF	Data Analysis	
[126]		TCGA PAAD	180	methy			
				CNV			
MOFA+		Private	Not provided	Multi-omics	NMF	Data Analysis	Python and R code
[130]							
iCluster	x	TCGA CRC	189	Exome sequence	NMF	Unsupervised Clustering	R code
[123]				mRNA		(Patient subtype identification)	
and iCluster+				methy			
[131]				CNV			
DFMF$^{3}$				GO terms	MTF	Unsupervised Clustering	Python code
[31]				GO annotations		(hepatotoxic risk associated	
[143]				Drugs		with individual drugs)	
				Tissue samples			
				DILI potentials			
MaDDa		TCGA BRCA,	200	Gene–gene interactions,	MTF	Unsupervised Clustering (Patient subtype identification)	Matlab code
[129]		BioGRID		Gene–pathway associations			
		KEGG		Disease–disease relationships,			
		Disease Ontology		Disease–gene associations,			
		DisGeNET		Disease–pathway relations			
DS-ICA	x	Private	38	features from EEG	ICA	Data Analysis	
[142]				and fMRI images			
MISA	x	Private	1001	EEG images	BSS	Data Analysis	MATLAB code
[21]				sMRI and fMRI images			

**BSS**: Blind Source Separation; **CNV**: Copy Number Variation; **DILI**: Drug-Induced Liver Injury; **EEG**: Electroencephalography; **fMRI**: functional Magnetic Resonance Imaging; **GO**: Gene Ontology; **ICA**: Independent Component Analysis; **KEGG**: Kyoto Encyclopedia of Genes and Genomes; **methy**: DNA methylation; **miRNA**: micro RNA; **MF**: Matrix Factorization; **mRNA**: messenger RNA; **MTF**: Matrix Tri-Factorization; **NMF**: Non-negative Matrix Factorization; **sMRI**: structural Magnetic Resonance Imaging; **TCGA+cancer code**: The Cancer Genome Atlas+ *link to complete cancer codes*.

The strategy applied by ’output-fusion’ methods is sketched in Figure 4 and their experimental design is summarized in Table 8. They apply individual PSN pipelines on each data source to obtain individual clustering or supervised prediction results (Figure 4**A** and **B**). All the obtained results are then fused by aggregation strategies that, acting as judges, compute a final decision by considering all the individual decisions taken by each unimodal pipeline.

**Table 8:** Output-fusion methods. For each method, the table reports: the name/acronym with the corresponding reference paper; the dataset used to develop and evaluate the approach in the reference paper and the corresponding sample cardinality and data types composing the dataset; the exploited integration method; the application task and the code availability (with link to the repository and programming languages for which the code is available)

Name	Dataset	Sample cardinality	Data type	Integration approach	Task	Code and Language
COCA	TCGA AML	161	DNA sequence	Consensus Clustering	Unsupervised Clustering	ConsensusClusterPlus
[22]	TCGA BIC	834	mRNA		(Patient subtype identification)	R code
	TCGA COAD	182	miRNA			
	TCGA READ	73	methy			
	TCGA GBM	195	CNV			
	TCGA KIRC	475	RPPA			
	TCGA LUSC	238				
	TCGA OV	329				
	TCGA UCEC	345				
	TCGA BLCA	120				
	TCGA LUAD	270				
	TCGA HNSC	305				
PINS/PINSPlus	34 TCGA datasets	12158	mRNA	Consensus Clustering	Unsupervised Clustering	R code
[23]	2 Metabric datasets		miRNA		(Patient subtype identification)	
			methy			
SUMO	TCGA extract	3168 across	mRNA	Consensus Clustering	Unsupervised Clustering	Python code
[62]	from NEMO		miRNA		(Patient subtype identification)	
	[94] (Table 4)		methy			
FH-Clust	TCGA AML	170	mRNA	Consensus Clustering	Unsupervised Clustering (Patients’ clusters related to known Survival)	R code
[24]	TCGA BIC	621	miRNA			
	TCGA COAD	220	methy			
	TCGA GBM	274				
	TCGA KIRC	183				
	TCGA LIHC	367				
	TCGA LUSC	341				
	TCGA SKCM	448				
	TCGA OV	287				
	TCGA SARC	257				
[145]	TCGA KIRC	418	Histopathological image	Stacked Generalization:	Supervised Classification	
	TCGA OV	250	RNA-seq data	linear regression of	(Cancer grade < 3 versus Cancer grade >= 3)	
	TCGA KIRC	220		unimodal classifiers	Supervised Classification	
	TCGA OV	160			(Known Survival < 5 versus Known Survival >= 5)	
[63]	ADNI Phase 1	628 for training	Demographic data	Average	Supervised Multiclass classification	Python code
	[155]	94 for validation	APOE e4 allele information		(HC versus MCI versus AD)	
	AddNeuroMed study	88 for testing	anatomical brain features			
	[156]		from 1.5T MRI scans			

**AD**: Alzheimer’s Disease; **ADNI**: Alzheimer’s Disease Neuroimaging Initiative; **CNV**: Copy Number Variation; **HC**: Healthy Control; **MCI**: Mild Cognitive Impairment; **methy**: DNA methylation; **miRNA**: micro RNA; **MRI**: Magnetic Resonance Imaging; **mRNA**: messenger RNA; **RPPA**: Reverse-Phase Protein Arrays; **TCGA+cancer code**: The Cancer Genome Atlas+ *link to complete cancer codes*.

### Input data-fusion via PCA-based and CCA-based methods

In the bioinformatics field, consensus PCA (CPCA [99]), hierarchical PCA (HPCA [106]) and Multiple Factor Analysis (MFA [107]), are some of the most used PCA-based integrative methods. They achieved interesting results on multimodal datasets including different types of patient data, from omics [18] to images [108–110].

Their effectiveness is due to their ability to project the data-blocks into a lower dimensional space spanned by not-correlated axis (principal components) maximizing the within-block variances and between-block covariances [111, 112]. By stretching the data along those axis, they induce a natural separability that improves the performance of the downstream algorithms, which are mostly devoted to data exploration and unsupervised clustering, though some exceptions using supervised clustering exist [20] (Table 6).

The difference between the three approaches relies on the way the latent space is found. Indeed, while ’CPCA’ solves an optimization problem by an iterative algorithm in the set of nonlinear iterative partial least squares methods (NIPALS [113]), ’HPCA’ [106] and ’*MFA*’ [107] consecutively apply PCA on respectively: (a) each block separately to derive lower-dimensional ‘stretched’ block representations maximizing the within-block variance; (b) the concatenation of the obtained block representations to derive a stretched latent space maximizing the between-block covariance.

A notable generalization of PCA for multimodal data is ’JIVE’ (Joint and Individual Variation Explained, [18]), which explicitly models each data-block $ {X}_i$ as the sum of a matrix representing the joint structure associated with ${X}_i$ and shared with other sources, and a matrix representing the source-specific structure characterizing $ {X}_i$, and residual noise. Given this formulation, authors apply an iterative estimation procedure that minimizes the reconstruction error, while constraining the axis of the joint and individual structures to be orthogonal (i.e. the joint and individual structures must be uncorrelated). In practice the estimation iterates over the following two steps: (1) having removed the individual structure, apply a sparse singular value decomposition (SVD) to estimate a lower-dimensional joint structure; (2) having removed the joint structure, apply a sparse SVD to find a lower-dimensional individual structure. Interestingly, JIVE also provides a permutation test to select the optimal ranks for the estimated structures. When experimented on multi-omics data from the glioblastoma multiforme (TCGA-GBM) dataset [18], JIVE showed its ability to effectively uncover the individual and joint data structures, thus leading to a better interpretation of interactions among data types and improving unsupervised classification results. Since the computational complexity of JIVE hampers its applicability, it has been recently reformulated (Angle-Based JIVE—aJIVE [97]) by using a hierarchical strategy similar to HPCA, which also produces more intuitive interpretations of the obtained decomposition, especially in the presence of strong collinearities. The effectiveness of aJIVE is witnessed by the promising results obtained when applied to an extract of the TCGA breast cancer dataset from [101] for the (supervised) task of tumor subtype prediction [114]. In particular the estimated joint components and the first five individual components for each data block are used to compose the integrated sample views to train Random Forest classifiers [115].

Opposite to PCA-based integrative models, Canonical Correlation Analysis-based (CCA-based) integrative models, e.g. Regularized Generalized CCA (RGCCA) [104, 105] and its sparse counterpart Sparse Generalized CCA (SGCCA) [19, 105], find the latent space maximizing the correlation within and between the different data-blocks. They are generally used for exploratory variable analysis since they try to bring all the data blocks to a unique distribution, therefore uncovering hidden relationships between different sources. However, DIABLO [20] has shown that SGCCA is also effective in the context of supervised clustering for patients’ subtype prediction. In practice, given a multimodal dataset containing $N$ samples organized into $Y$ classes, DIABLO firstly creates an extra dummy (supervising) data-block where each column is an indicator variable for the point-class ($1...Y$). Next, it uses SGCCA to maximize the covariance between all the data-blocks, including the supervising data-block. Given this representation, supervised clusters may be identified either (1) by averaging the components across data-blocks, to obtain an integrated patient representation that is then used by any supervised clustering algorithm (such as the Maximum Centroids algorithm [116]); (2) by applying the Maximum Centroids algorithm on each projected data-block to obtain individual clustering results, subsequently aggregated via a majority voting algorithm.

Though effective in several applications, all the aforementioned PCA-based methods suffer from two main limitations: sensitiveness to outliers and inability of handling missing data. Generalized Integrative PCA (GIPCA) [100] has been recently proposed as an extension of CPCA for dealing with missingness of some values and of entire views. To this aim, eigenvectors are used to explain the intra/inter-block variance by neglecting those samples/views with missing values/views.

### Input data-fusion via MF-based methods

MF methods [117] embed the points into a latent space that minimizes the reconstruction error and whose components (factors) are not constrained to be orthogonal (as in PCA) [31, 118, 119]. The most effective and used MF method applied on unimodal data is non-negative MF (NMF, [120]); it constrains both the component and loading matrices to be non-negative, which makes the approximation purely additive.

Given its effectiveness, several works proposed methods where NMF is extended to the integration of multimodal datasets (Table 7). The most relevant example is joint NMF (jNMF [121]) where multiple NMF problems are solved subject to a shared factor matrix that contains the basis vectors of the shared latent space. However, jNMF is sensitive to random noise and confounding effects [98] that are specific to each source, and that cannot be detected if a unique shared factor matrix is estimated. This affects the accuracy of the common structure estimation computed by jNMF [98]. Therefore, integrative non-negative matrix factorization (iNMF [98]) uses an approach similar to JIVE, where the factor matrices to be estimated are composed both by a shared and a source-specific structure. Unsupervised clustering experiments on the TCGA dataset [98, 122] have proven the superiority of iNMF with respect to jNMF [121], NMF [123] and to integrative Bayesian methods [124, 125].

Integrative Graph Regularized Non-Negative Matrix Factorization (NMF) for Network Analysis (iGMFNA [126, 127]) proposes improving the minimization of the reconstruction error, typical of NMF, by exploiting a graph view on each data block. Thanks to such representation, the designed iterative optimization minimizes the reconstruction error while maintaining the topology of the graph views. When compared with jNMF and iNMF to prioritize genes associated with cancer in two TCGA datasets by an unsupervised clustering approach, iGMFNA showed its superior performance.

The popular Penalized Non-negative Matrix Tri-Factorization (NMTF, [31, 128]) starts from a relational matrix ${R}_{1,2}$ containing non-negative elements that represent the strengths of the relationships between objects of two different types, $\epsilon _1$ and $\epsilon _2$, whose respective characteristics are defined by specific constraints, ${\theta }_1$ and ${\theta }_2$. NMTF finds the decomposition of ${R}_{1,2}$, subject to constraints ${\theta }_1$ and ${\theta }_2$, such that: $ {R}_{1,2} \approx{G}_1 {S}_{1,2} {G}_2^T$ so that ${G}_1$ and $ {G}_2$ are the low-dimensional representations of objects with types, respectively, $\epsilon _1$ and $\epsilon _2$, and $ {S}_{1,2}$ is the backbone matrix linking the two types.

NMTF is exploited by [31] in Data Fusion by Matrix Factorization (DFMF), where the reliability of the integrated low dimensional estimates computed over a multimodal dataset is improved by considering all the relational matrices (and corresponding constraints) linking the different sources between each other and with the patient data. Given all the relational matrices, ${R}_{i,j}$, and respective constraints, each ${R}_{i,j}$ is decomposed so that each backbone matrix represents the latent structure between two data types, the generic low-dimensional data representations of objects with a specific type, ${G}_i$, is bound to be used in the reconstruction of every relational matrix involving that type. Thanks to the abundance of information, the proposed model can also handle missing data and treat sparse relational matrices. Furthermore, it does not make any assumption about the structural properties of relations, which can also be asymmetric. DFMF can also be used in a semi-supervised setting. During training, the model parameters (i.e. the factorization ranks) are learnt, and are then used in a matrix completion problem, where unobserved entries in the target matrix ${R}_{i,j}$ are reconstructed for elements that were not present in the training set.

DFMF has been successfully used in the Matrix trifactorization for Discovery of Data similarity and Association (MaDDA) algorithm proposed by [129] to construct PSNs for unsupervised clustering. In particular, given $n$ patients to be partitioned into $k$ clusters, the low-rank matrix ${G} \in{R}^{n \times k}$ estimated through DFMF is viewed as a membership matrix relating each patient to the $k$ ranks/groups. After repeating the factorization multiple times with different initialization parameters, a final consensus matrix is obtained by element-wise averaging all membership matrices and then composing a PSN where the similarity between two patients (weight of the edge connecting them) represents how many times they ended up in the same group.

Multi-Omics Factor Analysis+ (MOFA+ [130]) is an integrative method exploiting Bayesian group factor analysis [51] with regularization to impose: (i) a view-wise and factor-wise sparsity, which shrinks to zero the loading for the $m$-th modality and the $k$-th factor if the latest does not explain any variability of the $m$-th view; (ii) a feature-wise sparsity, which sets to zero loading on individual features from active factors so that only a small number of features ‘actively’ contribute to each factor. MOFA+ can handle missing values as well as entirely missing views for some samples; moreover, it can cope with heterogeneous data types, which is exactly what is needed when dealing with multimodal datasets containing multi-omics, clinical and imaging data.

Given the successful results of MF-based integrative techniques, some authors have included them as a preprocessing step in their clustering/classification algorithms. As an example, iCluster+ [123, 131] uses NMF to fuse the heterogeneous data-blocks and then clusters the integrated views. It also exploits the obtained factor loadings to identify the relevant features in the cluster generation.

### Input data-fusion via BSS

In their original formulation, BSS models were defined as an extension of NMF techniques for ‘recovering unobservable source signals $s$ from measurements $x$ (i.e., data), with no knowledge of the parameters $\theta $ of the generative system $x = f(s, \theta )$’ [132].

Given their documented ability [132–134] of uncovering hidden structures underlying the observed unimodal signals, several BSS models have been extended to handle multimodal datasets comprising also ’multisets’ (Table 7), by a further step that estimates the mixing matrix that recombines all the estimated latent sources so as to compute an integrated, more informative signal with no redundancies [21, 132, 135, 136]. In particular, multisets are multimodal datasets containing multiple views acquired by the same source under different acquisition conditions (e.g. observation times, experiments, tasks, machines). They are therefore homogeneous [26] in semantic, type, and dimensionality. Multimodal-multisets are multimodal datasets acquired by different sources, among which sources producing multisets.

Given the lack of information about the mixing process and the source signals, BSS models often differ for the constraints they impose to counter the ill-conditioned problem and obtain essentially unique source estimates [132, 134, 137]. As an example, the well-known Independent Component Analysis model (ICA [138]), and its extensions to multimodal data (joint ICA—jICA [139, 140]), to multisets (Independent Vector Analysis—IVA [141]) and to multidimensional sources (Independent Subspace Analysis—ISA [142]), assume a linear (additive) mixture with mutually independent sources and a non-Gaussian distribution of each independent component in the latent space.

All the BSS models base their computations on the existence of collinearities between the observed multimodal data components, so that unreliable results may be obtained when this assumption is not satisfied. Some authors [135] circumvent this problem by preprocessing the data with CCA (or its multimodal extension), to obtain a projected data representation along correlated components.

The most representative BSS-based multimodal data integration technique is Multidataset Independent Subspace Analysis (MISA [21, 132]), which was recently proposed to generalize all the BSS models to the fusion of any kind of multimodal-multisets. Motivated by the definition of multiset, MISA is driven by statistical independence between latent subspaces while assuming correspondence within the subspaces underlying the input multisets. In practice, it firstly removes redundancies by estimating nonorthogonal demixing matrices, projecting each multiset into a respective (intermediate) lower-dimensional space spanned by independent components. The sources from all the computed latent spaces are then combined through another demixing matrix that brings all the data-blocks into a unique shared latent space, resulting in an integrated patient view. The de-mixing matrices are estimated by minimizing the mutual information in the final space, while maximizing the mutual information in the intermediate spaces, so as to capture as much correlation as possible. When applied to the integration of the information extracted from functional magnetic resonance imaging (fMRI), structural magnetic resonance imaging (sMRI) and electroencephalogram (EEG) data, MISA has proven its robustness with respect to high signal-to-noise ratios as well as its ability to produce effective data fusion in different ICA contexts.

### Output-fusion methods

Following Figure 4, in the context of multimodal PSN analysis the output-fusion methods described in this section may be applied to combine the (unsupervised clustering or supervised classification) results (Figure 4**B**) computed by individual PSN analyses applied on each data block (see Figure 4**A**). In Figure 4**C**, the combination of the unimodal results is performed either by some heuristics, or by majority voting, or by using a meta-model that learns from the predictions performed by each unimodal PSN analysis. Output-fusion techniques have been proposed for clustering samples (mainly from the TCGA datasets, Table 8) to identify patients’ subtypes [22, 23, 144] and for patients’ classification [63, 145] (Table 8).

As an example, in Cluster-of-Cluster-Assignments (COCA [22]), authors combine the clustering results individually obtained by NMF [146] on each of the six data types of the TCGA datasets. To this aim, the samples are coded into vectors composed of indicator variables representing the clusters they have been assigned in each modality, so that they can be reclustered according to those vectors by Consensus Clustering Plus [147]. Given the number of clusters $k$, Consensus Clustering Plus works on a consensus matrix (${CM}_k$) representing ‘the proportion of clustering runs in which two items are [grouped] together’ [148]. Given ${CM}_k$ an agglomerative hierarchical consensus clustering using distance of 1-consensus values is completed and pruned to $k$ groups that are returned as consensus clusters.

PINSPlus [23, 144] similarly exploits Consensus Clustering [148] for reaching the final partition. In practice, Perturbation clustering for data INtegration and disease Subtyping (PINS) starts by applying any classic unsupervised clustering algorithm (e.g. k-means) individually on each of the $M$-th datasets. If $n$ is the number of patients, for the $m$-th dataset ($m \in{1, \dots , M}$) the clustering result is expressed by a square matrix ${C}_m \in \mathbb{R}^{n \times n}$, such that ${C}_m(i,j)=1$ if samples $i$ and $j$ fall in the same cluster, and ${C}_m(i,j)=0$ otherwise. All the resulting matrices are then averaged to obtain a consensus matrix ${S} = \frac{\sum _{m = 1}^M {C}_m}{M}$. Even though matrix ${S}$ may highlight that some points do not reach a strong agreement, authors consider that ${S}$ itself may be used as a pairwise similarity matrix (since ${S} = 1$ for points for which there is a strong agreement, viewed as similarity, across all the dataset, and ${S} = 0$ otherwise) that is suitable for similarity/distance-based clustering algorithms such as any hierarchical Clustering algorithm [149], Partitioning Around Medoids [150] or dynamic tree cut [151]. In their work, authors propose testing different clustering algorithms and then choose the partition that agrees the most with the partitioning of individual data types.

Consensus clustering has also been successfully applied by the recently published SUMO [62], an integrative clustering algorithm that starts by computing several unimodal PSNs by using a scaled-normalized Euclidean kernel similar to the one exploited by SNF [3]. SUMO then formulates a constrained NMTF (see Section Input data-fusion via MF-based methods) to find a sparse shared representation of all the samples in the cluster subspace by accounting for the adjacencies observed in all the data types. The NMTF optimization problem is solved by an iterative procedure that is applied several times on several sample subsets to ensure robustness with respect to the initial conditions and to the input data; consensus clustering is then exploited to pool together the clustering results. When compared with the most promising integrative clustering methods (e.g. iCluster [152], Multiple Canonical Correlation Analysis (MCCA) [102], NEMO [94], SNF [3], PINSPlus [23]) SUMO obtained impressive results.

The Fuzzy-hierarchical CLUSTering—FH-Clust method [24] interestingly proposes to use fuzzy logic for identifying patients’ prognostic subgroups from multiomics data, resting on the fact that in nature there is often no clear cut between subtypes. Unimodal data are separately analyzed using a fuzzy-based hierarchical clustering approach exploiting a Lukasiewicz valued fuzzy similarity and individual results are then fused through a consensus matrix. Extensive experiments on 10 cancer datasets from TCGA (considering gene expression, miRNA, methylation data) show that FH-Clust is competitive with state-of-the-art methods (i.e. k-means, Spectral Clustering, LRACluster, PINS, SNF, MCCA).

Interesting output-fusion approaches aimed at patients’ classification are described in [63, 145]. In [145] the authors obtain effective cancer-grade and patient-survival classifications for cancer patients represented in the TCGA renal (TCGA KIRC) and TCGA ovarian (TCGA OV) datasets by using all the data types included in TCGA, including hematoxylin and eosin (H&E)-stained whole-slide images of tissue samples that are processed by digital image processing techniques to extract more that 400 features per sample. In practice authors firstly individually process each data block to apply an internal cross-validation approach to choose (1) the number of informative features to be extracted by the minimum Redundancy Maximum Relevance (mRMR) method [153] and (2) the best performing 5-fold cross classifier among SVM, logistic regression, K-nearest neighbors and Linear Discriminant Analysis. To compose all the predictions from the different modalities authors compare the stacked generalization model [154], which essentially trains a logistic regression classifier on the obtained predictions, to the majority vote strategy. The best results are obtained by the stacked prediction model, which leverages the results obtained by any of the multimodal predictions, independent from the classifier that is used for producing them.

In [63] authors simply use the average to integrate the different prognostic classifications computed over multimodal profiles of suspected Alzheimer Disease (AD) patients, with the aim of identifying patients who are vulnerable to conversion from mild-cognitive impairment to AD. In particular, the squared-exponential kernels are firstly used to build unimodal PSNs, and, for each unimodal network, a Gaussian process is then exploited to assign labels to unknown points based on the nearest known points. Finally, the unknown patients’ condition is computed as the average over all the unimodal predictions.

## Discussion and conclusion

In the context of precision medicine, PSNs are gaining momentum given their ability to uncover and exploit relationships among patients when applied to clustering and classification tasks [9]. According to the state-of-the-art surveys describing the application of PSNs for precision medicine or health data processing [9, 45, 157, 158], PSN-based models benefit from several advantages; they are: (1) easy to understand, (2) interpretable by design, (3) privacy preserving, (4) competitive or even superior to state-of-the-art clustering/classification methods, (5) potentially able to integrate different data views. In particular, the possibility of using PSN models in a multimodal setting is especially relevant in light of the increasing availability of digital technologies by means of which huge amount of multimodal data can be collected that describe each patient/sample by considering different biological/medical views. Moreover, in the past few years the increasing availability of cloud technologies allowed us to distribute data processing across multiple local servers belonging to, e.g. different institutions. In this context, the development of promising information integration models would allow the application of a Federated Learning strategy [159], where a central server collects, further integrates, and eventually processes, the (already) integrated data, or the individual PSNs, or the predictions individually computed by local servers located in the institutions where the data belong. In this way, the initial processing of the sensitive data would be demanded to the local institutions to protect patient privacy, and the central server would have access only to preprocessed information, thus hiding explicit sensitive data.

Though in the biomedical context several multimodal approaches have already shown their ability to integrate multimodal data to improve the results obtained from a single view (unimodal data) [114], and the survey literature about data integration methods for multimodal data is wide [13, 15, 16], in the field of PSN analysis only few methods have already investigated the usage of multimodal data, by building integrated PSNs that exploit both the joint and the individual information from all the available sources. Moreover, no state-of-the-art survey has focused on the role of PSN as a cornerstone for data fusion. In this survey, we aim at filling this gap with the goal of providing interested readers with a comprehensive collection of integrative methods that may be exploited to build PSN approaches efficiently handling multimodal data.

Besides an extensive literature search, the integration approaches have been organized into three broad classes on the basis of the type of data that is fused: ’PSN-fusion’, ’Input data-fusion’ and ’Output-fusion’ methods. More precisely, ’PSN-fusion’ methods may be split into the three sub-classes of ’MKL’, ’SNF-based’ and ’other’ methods, whereas ’Input data-fusion’ approaches comprehend algorithms ’PCA-based’, ’CCA-based’ and ’MF-based’.

The survey has highlighted the promising results and advantages that characterize the methods belonging to the three classes of our proposed taxonomy.

Methods based on PSN-fusion techniques are particularly useful in network medicine applications [160] that study human diseases through ‘systemic’ approaches in which diseases are interpreted as perturbations in complex biomolecular networks. In this context, transductive strategies working on individual PSN models [7] would benefit from the application of PSN-fusion approaches, as shown by recent promising results [3, 6].

Methods based on input-data fusion techniques rely on factor analysis models for the removal of data collinearities and the simultaneous enhancement of the individual structure characterizing each view. For this reason, we believe such techniques are particularly useful when dealing with multiview data involving follow-up examinations, where the multiple views likely contain correlated information.

Output-fusion techniques should be used when the differences between the multimodal views impose the usage of peculiar and specific unimodal PSN models for obtaining individual inferences. This is the case, for example when we need to combine data having substantially different structures, ranging from vectorial to sequence and graph-structured data.

Though being effective, our thorough review also evidenced difficulties and drawbacks that harbour from the data-fusion strategy. In particular, PSN-fusion models require to build an individual PSNs on each data type. This raises the crucial, still open, and often overlooked problem of choosing proper individual similarity measures for building each unimodal PSNs. Indeed, only few methods [60, 161, 162] reported exhaustive comparative evaluations among few distance metrics applied to genetic data. By considering that several problems in precision medicine are characterized by nonlinearly separable omics data, and given the experimental results we have collected during our literature search, we recommend computing PSNs by exploiting a kernel function. In this context, though several functions have been successfully proposed and used in literature, when dealing with continuous data, we suggest using the scaled exponential kernel of Euclidean distance [3, 62], due to its ability to adapt to different neighborhood sizes. This allows dealing with datasets distributed on complex manifolds where datapoints are not evenly distributed in space, as it often happens in real-world problems. On the other hand, when dealing with simpler data types with lower dimensionalities and complexities (e.g. clinical data), simpler normalized similarities may be sufficient to appropriately capture the data structure. Clinical datasets usually contain categorical variables, often mixed with numeric features. The former situation can be appropriately addressed by averaging the normalized similarities individually computed on each variable [6], whereas Chi-squared distances are the most suitable for categorical data [3, 6]. Of note, the subsequent application of a Random Walk kernel, as proposed by Gliozzo *et al*. [7], is a promising step to refine the obtained PSN.

On the other side, input data-fusion techniques integrate the input data by projecting them into a shared space with lower dimensionality, thus making these approaches strongly dependent on the chosen final dimensionality $d$.

While classic approaches have been proposed to automatically set $d$ [100, 163–165], this value is often user-defined after observation of the scree plot. However, observing that the optimal latent vector space is the one that allows to capture the intrinsic data structure, we instead suggest setting $d$ to the intrinsic data dimensionality (id) [166], which is the minimum number of parameters needed to represent the data without information loss.

Finally, output-data fusion methods are often too generic or use very simple output-aggregation strategies, e.g. average or majority voting, that may produce suboptimal results.

Generally speaking, our survey evidenced some important open issues in the context of data integration methods for PSN that call for the future research directions summarized in the following subsection.

### Future research directions

While conducting our survey we noted the need of investigating methods for data pre-processing, with the aim of, e.g. detecting and eliminating noise with heterogeneous characteristics, collinearities between different views, and confoundings that could bias the final results (as per [27]). Indeed, only few recently proposed preliminary attempts were able to explicitly consider the presence of noise with heterogeneous characteristics [98, 122].

Moreover, future research should be devoted to the investigation of novel multimodal feature-selection algorithms. Indeed, the few methods applying a feature selection step exploit either classic univariate statistics, or algorithms, such as mRMR [153], that analyze group of features by neglecting their multimodal characteristics.

On the other side, missing data imputation needs deeper investigation to handle two types of biomedical data-missingness: (1) missingness of some data values in some views; (2) missingness of entire views for some samples. While missingness is becoming a common problem in different fields, in the biomedical field few approaches present thorough missing data imputation studies [11]. Besides, among the approaches we have surveyed, only GIPCA [100] specifically addressed both these types of missingness. Finally, given the big-data produced by high-throughput technologies, scalability is becoming an important and often overlooked issue, nowadays hampering the applicability of several promising tools.

Though the aforementioned issues are still open, all the surveyed strategies have reported promising results that might improve knowledge in then field of precision medicine. Unfortunately, different similarity metrics, experimental setups and evaluation measures are used for model assessment; this hampers an objective comparison between the different integration techniques and data analysis models. Furthermore, we found no evidence about data integration approaches that should be preferred over the others. Instead, the type and semantic of the available data type and the specific biomedical question to address should guide the choice. An additional open problem regards the identification of the most appropriate similarity/distance measure for each biological data modality. To the best of our knowledge, only few works tried to investigate this issue by comparing different metrics for specific data views and most of them are focused on gene expression data [60, 161, 162]. Comprehensive studies comparing the usage of different similarity measures in different contexts (e.g. when applied to different biological data types and in supervised and unsupervised prediction contexts) would provide fruitful insights to guide the scientific community towards effective PSN construction. We also remark that, though some algorithms are already available as open source packages/repositories (mostly coded using R, Python and Matlab) [16], many others are not, thus slowing down their diffusion and testing by the community.

Another interesting research line that should be given attention is represented by the development of Web applications extending, e.g. those presented in [167, 168], for the visual analysis of PSN models. Indeed, the graphical tools can enable the visual comparison of different PSN models realized according to any of the methods discussed in this survey. This in turn can improve the explainability of the computed results and would allow the user to choose the approach mostly suited to her/his needs.

## Author contributions statement

J.G., G.V. and E.C. conceived the work, J.G. collected the literature papers, J.G. and E.C. studied the literature, selected the most relevant works and drafted them; J.G, M.M., M.N., A.Pac., G.V., E.C. wrote the paper; all the authors validated the work.

**Key Points**

- Patients similarity networks (PSN) are explainable and privacy preserving representations of patients that leverage the similarity of their clinical/biomolecular profiles to construct graphs of patients.
- Network Medicine algorithms on PSNs for patient stratification, phenotype and outcome prediction and disease risk assessment represent novel tools for Genomic and Precision Medicine
- The combination of clinical, omics and imaging bio-medical data can lead to novel PSNs able to leverage the synergy of multiple views of the patients.
- Several reviews about data integration methods in bioinformatics and biomedical applications have been proposed but no specific reviews about the emerging field of heterogeneous data integration methods for patient similarity networks are actually available.
- We provide a thorough review and propose a taxonomy of heterogeneous data integration methods for PSNs, together with the different patient similarity measures proposed in literature.
- We also review methods that have appeared in the machine learning literature but have not yet been applied to PSNs, thus providing a resource to navigate the vast machine learning literature existing on this topic.
- Strengths and limitations of the proposed methods are discussed to both assist researchers in the design and development of novel methods and to guide the selection of PSN integration methods for specific applications, focusing on methods that could be used to integrate very diverse datasets, including multi-omics data as well as data derived from clinical information and medical imaging.

## Author Biographies

**Jessica Gliozzo** is a PhD student in computer science enrolled in the Collaborative Doctoral Partnership Program between the University of Milan and the Joint Research Center of European Commission. Her latest research works comprehend the development of a multi-modal semi-supervised method based on patient similarity networks for patients’ outcome prediction; the application of deep neural networks to predict the tissue-specific activity status of *cis*-regulatory regions in the genome (i.e. promoters and enhancers); the use of compression methods to obtain compact representations of convolution neural networks in the biological domain (e.g. ki67 and TIL-index prediction), showing their advantages when limited computational resources are available. She is author of a few works in the fields of machine learning and bioinformatics.

**Marco Mesiti** is associate professor at the Department of Computer Science Giovanni degli Antoni, Universitá degli Studi di Milano. He has got a master and PhD degree from the University of Genova in 1998 and 2003. His research interest are in the integration, querying and visualization of different kinds of information (structured and semi-structured) according to different data models (relational, graph and nosql). Moreover, he has involved in different projects for protein network integration, protein function prediction and protein networks visualization. On these topics he has published > 100 articles in international conferences and journals. He is associate editor for the Springer Data Science and Engineering Journal and MDPI Applied Sciences.

**Marco Notaro** is a postdoctoral fellow at the Computer Science Department of Milan University. His research interests touch the fields of bioinformatics, computational biology, biological network and machine learning. His main expertise is the analysis and construction of complex biomolecular networks and the design and implementation of output-structured learning algorithms to discover novel gene–disease associations or to predict novel protein function. His PhD paper was awarded by International Medical Informatics Association as one of the best five papers of 2017 in the field of medical informatics.

**Alessandro Petrini** is a postdoctoral researcher at the Department of Computer Science of Universitá degli Studi di Milano. He is currently a member of the Laboratory of Bioinformatics and Computational Biology—AnacletoLab—and his main research is focused on high-performance computing and machine learning. He is author of > 30 articles in international journals and conferences. He designed and developed parallel and accelerated ML algorithms for image and video processing/encoding/compression, omics analisys, graph modeling and analysis, data visualization, neural network compression, MRI volumes processing and analysis.

**Alex Patak**, PhD, MD, graduated in medicine and surgery at the School of Medicine at ‘Universidad Autónoma de Barcelona’, Barcelona (Spain) and holds a Master in medical bioengineering from the ‘Universidad Politécnica de Cataluña’. At the Instituto Municipal de Investigación Médica (Barcelona) he has been working on expert systems for medical diagnostic and did his PhD on computer-assisted medical education after a stage at Dartmouth Medical College in Vermont (USA). Since 1994 works at the Joint Research Centre in Ispra (Italy) where he has been working on three-dimensional medical imaging, and from 2003 to 2017 was responsible for the bioinformatics team at the Molecular Biology and Genomics Unit of the Institute for Health and Consumer Protection in Ispra. He is now a team leader at Knowledge for Health & Consumer Safety and is responsible for the Collaborative Doctoral Partnership Programme in Genomics and Bioinformatics, working on the application of artificial intelligence to omics data and microbiome.

**Antonio Puertas-Gallardo** is an IT project manager at the Joint Research Center (JRC) of the European Commission. He provides high-performance computing (HPC) support to bioinformatics members of the Knowledge for Health and Consumer Safety Unit at JRC, and he has recently begun to collaborate with the unit’s data scientists on natural language processing and machine learning.

**Alberto Paccanaro** is full professor in machine learning and computational biology at the School of Applied Mathematics of the Fundação Getúlio Vargas in Rio de Janeiro and at the Department of Computer Science at Royal Holloway University of London, where he is also Director of the Centre for Systems and Synthetic Biology. He completed his undergraduate studies in computer science at the University of Milan and received his PhD from the University of Toronto in 2002. His research interests are in applying and developing machine learning algorithms for solving problems in molecular biology, medicine and pharmacology and he has led a number of international research projects in this area.

**Giorgio Valentini** is a full professor at the Department of Computer Science, University of Milan (UNIMI). Director for UNIMI of the European doctorate in Genomics and Bioinformatics in collaboration with the Joint Research Center of the European Union. Director of AnacletoLab, Computational Biology and Bioinformatics Laboratory of the Department of Computer Science of the University of Milano. He has been PI in several national and international research projects funded by public and private institutions in the area of bioinformatics, machine learning and big-data analytics. He is author of over 150 scientific publications with peer-review in collaboration with several research groups in Europe and America in the field of bioinformatics, computational biology and machine learning.

**Elena Casiraghi** is associate professor at the Department of Computer Science Giovanni degli Antoni, Universitá degli Studi di Milano. She is co-lead of the AnacletoLab, Computational Biology and Bioinformatics Laboratory of the Department of Computer Science of the University of Milano. Her research interests are focused on the development of applications that process biomedical data (images, multi-omics, clinical) for causal inference, inductive inference, machine learning and pattern recognition. She currently cooperates with several hospitals and research centers in Italy (Istituto Nazionale dei Tumori, Ospedale Policlinico e Regina Margherita, Ospedale San Raffaele, Humanitas), Europe (Berlin Institute of Health at Charité, grupo espanol de investigacion de sarcomas, groupe sarcomes francais) and United States (Berkeley Laboratories, Jackson Laboratories) and she is the scientific supervisor for UNIMI of all the researches in cooperation with the N3C Enclave (USA, https://covid.cd2h.org/) created and funded by the NIH.

## Appendix A - Data integration in Medicine: previous surveys and taxonomies

The abundance of multimodal data integration approaches developed in the past decade in the biomedical context has motivated many relevant surveys [16, 29, 34–37], which proposed different definitions and taxonomies, schematized in Figure 1.

In the context of precision medicine, multimodal sets are composed of multiple views (or data-blocks) for the same set of patients. They are either ’multisets’ or ’multimodal datasets’ [142]. ’Multisets’ (top of Figure 1-yellow box) contain multiple views acquired by the same source under different acquisition conditions (e.g. observation times, experiments, tasks, machines), and are therefore ’homogeneous’ [26] in semantic, type and dimensionality. Conversely, ’multimodal datasets’ (alias ’heterogeneous’ sets [26], Figure 1-light blue box) contain data-blocks acquired by different sources, characterized by different semantics, type and dimensionalities. Among the latter, ’multimodal-multiset’ are datasets acquired by different sources, some of which are used to produce multisets.

Given their different characteristics, multisets and multimodal datasets are generally fused by following different integration flows. ’Horizontal integration’ methods [25, 169] are usually used for multisets because they equally process all the data-blocks and then pool the obtained results by e.g. summary statistics [170]. By contrast, ’vertical integration’ methods are used for processing multimodal datasets, which are more articulated and are usually grouped in the ’hierarchical-vertical’ class and the ’parallel-vertical’ class.

’Hierarchical-vertical’ [171–173] or ’multi-staged analysis’ methods [27] consider omics data being interrelated by regulatory mechanisms and exploit such prior knowledge during the integration procedure. Since these methods are tailored for the treatment of specific data types and applications and cannot be generalized to different research contexts, they will not further considered in this survey. ’Parallel-vertical’ integration techniques, alias ’meta-dimensional analysis’ methods [27], are the most diffused and generalizable ones because dependencies between data-blocks injected by prior information are not considered. To categorize parallel-vertical approaches several interrelated taxonomies have been defined. The categorization reported in the red-dashed box in Figure 1 is the one adopted by several authors [27, 29–33] that relies on the processing stage (early, intermediate, late) in which the data fusion happens, which also influences the kind of information that is fused.

’Early integration techniques’ (also called ’concatenation-based models’ [27, 33]) are applied on the input data-blocks in an early stage to compose the integrated input vectors subsequently used in the analysis by either using a simple data-concatenation, or by exploiting joint latent space estimation models. The evident advantage of early methods relies on their ability to uncover the individual information characterizing each of the different sources as well as the hidden relationships between them. Another advantage is brought by the fact that early methods solve the integration problem in the first stage, so that any unimodal analysis process may be subsequently applied.

’Intermediate integration approaches’ (also named ’transformation-based models’) [27, 33] individually transform the data-blocks into intermediate (unimodal) models that are subsequently integrated to produce a unique fused model to be analyzed. In the taxonomy proposed by [28] (blue-dashed box in Figure 1), these methods have been classified as ’model-dependent’ approaches for highlighting their dependency from the data analysis model, which guarantees the ability to retain the original data structure by explicitly addressing the fusion task in the construction of the predictive model itself.

’Late integration approaches’ (also named ’model-based approaches’ in [27, 33]) separately analyze each of the incoming data-blocks to produce individual results, subsequently integrated in a late phase by some meta-learner acting as the final judge or by simple techniques such as majority voting. These approaches along with the early integration ones are classified as ’model-agnostic’ in the taxonomy proposed by [28] (blue-dashed box in Figure 1) and are contrasted with the model-independent approaches previously discussed. They are named “agnostic” because they are independent from the specific algorithm applied in the preceding unimodal analysis, which can be therefore tailored to the processed type.

Even if the aforementioned early/intermediate/late taxonomy is the most diffused in literature, other taxonomies have been defined in the context of integrative (multi-omics) methods for precision medicine. As an example, [25, 174, 175] consider three classes: (1) ’statistical-based methods’, most of which can be considered instances of the class of early integrative methods; (2) ’unsupervised methods’ neglecting the outcome variable during the integration phase, which may be applied in any (early, intermediate or late) phase and are mainly devoted to unsupervised clustering; (3) ’supervised integration methods’ fusing the available information to maximize the outcome prediction performance by mainly using an intermediate MKL integration approach or a late fusion approach.

Other taxonomies [8, 14, 16, 32] consider the specific algorithm used for the integration; they recognize network-based approaches (among which deep-network based approaches, not treated in this survey), feature transformation models mainly applying an early integration approach (e.g. PCA, CCA), integrative models exploiting MF techniques in an early integrative fashion, MKL models belonging to the class of intermediate methods, and Bayesian techniques applied in an early phase. Note that Bayesian models are not considered in this work since they have been exhaustively described in a dedicated survey [13].

Finally, the relevant survey by [16] is focused on the description of publicly available multimodal datasets in the context of multi-omics and in the critical analysis of open source integrative models. After a thorough study, the authors conclude that an objective comparison between different models is difficult, and highlight the lack of an easy-to-use multi-omics data fusion model providing a ‘biologist-friendly’ visualization and interpretation.
